# Insulator Abnormal Condition Detection from Small Data Samples

**DOI:** 10.3390/s23187967

**Published:** 2023-09-19

**Authors:** Qian Wang, Zhixuan Fan, Zhirong Luan, Rong Shi

**Affiliations:** 1 School of Electrical Engineering, Xi’an University of Technology, Xi’an 710048, China; 2221920078@stu.xaut.edu.cn (Z.F.); luanzhirong@xaut.edu.cn (Z.L.); 2 State Grid Shaanxi Electric Power Company Economic Research Institute, Xi’an 710065, China; shirong@jyy.sn.sgcc.cpm.cn

**Keywords:** insulator detection, electric power inspection, small sample data expansion, vision sensors, YOLOV5

## Abstract

Insulators are an important part of transmission lines in active distribution networks, and their performance has an impact on the power system’s normal operation, security, and dependability. Traditional insulator detection methods, on the other hand, necessitate a significant amount of labor and material resources, necessitating the development of a new detection method to substitute manpower. This paper investigates the abnormal condition detection of insulators based on UAV vision sensors using artificial intelligence algorithms from small samples. Firstly, artificial intelligence for the image data volume requirements was large, i.e., the insulator image samples taken by the UAV vision sensor inspection were not enough, or there was a missing image problem, so the data enhancement method was used to expand the small sample data. Then, the YOLOV5 algorithm was used to compare detection results before and after the extended dataset’s optimization to demonstrate the expanded dataset’s dependability and universality, and the results revealed that the expanded dataset improved detection accuracy and precision. The insulator abnormal condition detection method based on small sample image data acquired by the visual sensors studied in this paper has certain theoretical guiding significance and engineering application prospects for the safe operation of active distribution networks.

## 1. Introduction

An active distribution network is an important means to improve the ability of new energy consumption, improve the quality of electricity consumption and reliability of power supply, and realize the goal of double carbon, which is the development direction of future distribution networks. Multisource data sensing technology is one of the key technologies of an active distribution network, and it is also an important support for the safe operation of an active distribution network. In an active distribution network, the failure of transmission lines significantly affects the reliability and safety of the power supply. The insulator plays a crucial role in controlling insulation—it prevents the current from returning to the ground and supports the wire in the transmission line. Failure of the insulator can have severe consequences that directly impact the safe operation of the transmission line. Insulators are vulnerable to natural factors such as lightning strikes, snow, ice, high and low temperatures, as well as fouling and bird damage due to their long-term exposure to atmospheric conditions. All these factors can cause various types of insulator failure.

**Figure 1:**
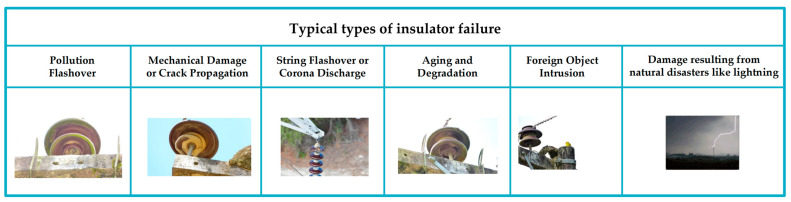
Typical types of insulator failure.

Several typical types of malfunctions exist that can result in insulator failure. Pollution flashover: When the surface of an insulator is contaminated by polluting substances, its insulating property decreases, making it more vulnerable to corona discharge and flashover. Mechanical damage: External forces such as impact, shock, vibration, or others may cause insulators to break, crack, or be damaged. String flashover, also known as insulator string jump flashover, can occur when there is an insulator chain between multiple insulators and the insulation performance of the insulator is not uniform. In such instances, a high-voltage string jump flashover phenomenon can lead to arc discharge. Corona discharge: When subjected to high voltage, air near the insulator may experience corona discharge that leads to corona sound and erosion of the insulator surface. Insulator breakdown: When voltage exceeds the insulating capacity of insulators, they may break down and result in short circuits or equipment damage. Crack propagation: Cracks may exist on the surface or inside the insulator, which, when gradually expanding under the influence of electric loads and the external environment, could lead to insulator failure. Aging and degradation: Over time, the insulating material ages and deteriorates because of various factors. Foreign object intrusion: External objects, such as birds, leaves, debris, and so on, could affect the performance of the insulator by entering the gap of the insulator. Insulator failure types are presented in Figure 1.

Insulators can be seriously damaged due to prolonged exposure to light and wind, leading to power system failures that affect the entire grid and cause financial losses. Therefore, insulators must be monitored. The online mode is also used to improve the efficiency of fault detection. Vision sensors and image sensors, along with their processing technology, can detect insulator faults using images captured by UAV inspections and network training. This reduces manual work pressure, improves efficiency, and enhances the safety of the inspection process.

The current insulator fault detection technology mainly has two difficulties: (1) the traditional insulator detection method requires a lot of human and material resources and is less safe, and (2) when using image recognition technology for detection, there is insufficient sample data, and technical algorithms are not comprehensive. Therefore, it is necessary to study the insulator abnormal state detection algorithm from small data samples.

During insulator detection, a limited number of images are captured using drones and other methods. Small datasets may not provide sufficient support for system analysis and identification, affecting identification accuracy. This is a common practical engineering problem. This paper proposes a solution to address actual problems in the power system based on this assumption and starting point. This study focuses on using images obtained from UAV vision sensors for inspection purposes. The insulators captured in these images are then used as detection targets.

This paper emphasizes the detection of abnormal status for insulators and uses a data enhancement method to simulate shooting situations under various scenarios. The simulation includes rotation, mirroring, cropping, changing brightness, adding noise, and more. Through the insulator images taken by UAV patrol, the database is expanded for its characteristics of complex shooting situations and the small number of photos taken. The insulators, including the normal insulator, defective insulator, bird’s nest, and grass, among other objects, are labeled with image features using LabelImg. Then, the YOLOV5 algorithm is used to train the model, enabling easy detection and classification of the insulator in the image.

The main contributions of this paper are in the following three areas: Firstly, this paper proposes an effective data preprocessing method for the specific task of insulator anomaly detection with a small sample dataset to improve the performance and generalization of the model through data augmentation. Secondly, we constructed a dataset for insulator defect detection, comprising defective and normal insulators. The photographs of varied insulator types under different angles and light conditions were simulated through data enhancement. It has a significant practical value. Thirdly, a fault detection model was trained using the YOLOV5 algorithm on a self-constructed dataset to solve real power system problems with limited sample data. Fourthly, this work aims to address the requirements of insulator testing and solve the problems associated with power system insulator testing. It is more user-friendly for application in the power system and addresses the actual needs.

The experimental results can prove that in the insulator anomaly detection method, based on the currently popular YOLOV5 algorithm, the detection of an insulator anomaly can be basically achieved. Moreover, for the case of a small dataset, the data enhancement for small sample data can improve the accuracy of the trained model to a certain extent, improve the robustness of the model, and improve model performance.

The remainder of the paper is divided into five parts: Section 2 gives related work. Section 3 introduces the method for insulator detection from small data samples. Section 4 shows the experimental results and analysis. Section 5 concludes the paper.

## 2. Related Work

In recent years, few sample image classification algorithms have developed rapidly. In terms of traditional image processing methods, Muhammad Tariq et al. [1] used morphological techniques to detect and identify insulators in power distribution systems. By performing morphological filtering, edge detection, and connectivity analysis on the images, insulators can be accurately detected and identified; Xiaobo Wang et al. [2] proposed a method for insulator detection in power transmission lines using computer vision techniques in 2017. Automatic detection and localization of insulators were achieved by image processing techniques, including image preprocessing, binarization, morphological operations, and connected region analysis; Alaa H. Hussein et al. [3] used digital image processing techniques for insulator detection in high-voltage transmission lines. Using image preprocessing, edge detection, and morphological processing, the insulators were successfully detected, and their location and shape information was extracted.

Vision sensors have developed rapidly in recent years and are widely used in digital cameras, smartphones, surveillance cameras, robotic vision systems, etc. With the development of technology, they have gone through several stages of development and improvement, such as CCD, CMOS, BSI, etc. Dlugosz et al. [4] proposed a CMOS image sensor with stacked pixels and pipelined Analog-to-digital conversion (ADC) sensors provide higher image quality and faster data conversion. For example, Akkermans et al. [5] introduced a backside-illuminated CMOS sensor that provides better image quality in low-light conditions by using backside illumination. Girshick et al. [6] proposed a real-time target detection method called the faster R-CNN detection method and introduced a region suggestion network to generate candidate target frames through end-to-end training and feature sharing. The faster R-CNN was able to achieve faster target detection while maintaining high accuracy. In 2012, Khoshelham et al. [7] explored the accuracy and resolution of Kinect depth data for indoor mapping applications through experimental evaluation and analysis, providing an in-depth understanding of the performance of Kinect depth data and discussing its potential for indoor mapping applications. In 2006, C. C. Ko [8] proposed an insulator fault detection and diagnosis method based on a computer vision system to detect abnormal insulator conditions through image processing and analysis.

According to different modeling approaches, existing algorithms can be divided into two main categories: convolutional neural network models and graph neural network models. Among these, migration learning is a learning method that takes what has been learned once and simply applies it to a new task. Matthew Zeiler and Rob Fergus et al. [9] first explored the problem of migration learning in deep neural networks in 2014; Christian Szegedy et al. [10] used integrated learning to combine multiple classifiers for classification in 2015 and proposed the inception network structure; Ian Goodfellow et al. [11] first introduced the concept of GAN, which can be used to increase the amount of training data in the field of image recognition, in 2016; and Chelsea Finn et al. [12] first introduced the concept of metalearning, which can be used to train a classifier that quickly adapts to new categories, in 2017. Based on these two findings, Egor Zakharov et al. [13] proposed a method that combines metalearning and GAN, which can be used to generate high-quality synthetic images.

With the development of intelligent technology and the popularization of civil aviation equipment, mechanical inspection of power cables has become possible. In the mid-20th century, several countries, led by the United States, experimented with the use of helicopters to inspect power networks. In 2008, the Universidad Politécnica de Madrid developed a vision system based on this, using an unmanned aircraft as a platform for collecting information [14]. The European airline Laserpas entered a partnership with Neurala Artificial Intelligence in 2018 to improve unmanned power monitoring operations based on the latest developments in artificial intelligence [15]. French electricity company Delair used LIDAR technology on an unmanned aircraft to create a 3D map of the electricity network from aerial imagery [16].

Fault detection techniques for insulators have also developed rapidly in recent years and can be broadly divided into four directions: In 2019, Xinjie Wang et al. [17] proposed a migration-learning-based fault detection method for insulators, which can use an existing large sample dataset for training and then apply the trained model to a new small sample dataset for fault detection. Zhiyu Cui et al. [18] proposed a deep-learning-based insulator fault detection method in 2010, which uses convolutional neural network (CNN) for insulator image feature extraction and classification for fault detection. Jin Zhang et al. [19] proposed a metalearning-based insulator fault detection method in 2021, which can learn on small sample datasets and quickly adapt to new small sample datasets for fault detection. Liu et al. [20] proposed the MTI-YOLO network, which used a multiscale feature detection head, a multiscale feature fusion structure, and a spatial pyramid pool model, which in turn improved the accuracy of the model, but its speed was slightly reduced, and it only detected normal insulators and did not develop detection for insulator defects. Singh L et al. [21] used thermal imaging to obtain porcelain insulator temperature distribution images. These images provide detailed information about the insulator surface temperature. These images are then analyzed and processed by machine learning algorithms to identify potential health problems or signs of defects.

This study proposes to use a data augmentation approach to expand the constructed small sample dataset and train an optimized model based on the YOLOV5 algorithm to effectively improve the accuracy and robustness of the model for identifying and detecting insulator states in the study images.

## 3. Proposed Insulator Defect Detection Method Based on Small Sample Data

This work is based on the YOLOV5 algorithm and uses a cloud-based hard drive to train and test the dataset.

### 3.1. LabelImg-Based Data Preprocessing

In this paper, LabelImg is used to annotate feature regions in images.

(1) By organizing the original dataset, it was divided into 120 normal insulators and 120 defective insulators, and the labeled contents were divided into five categories by the software: Defective Insulator, Nest, Grass, Bird, and Normal Insulator.
(2) The different types and colors of the boxes were used to distinguish them and to obtain the label file needed to use the YOLO algorithm. For YOLOV5, the more practical label file was the txt file. Therefore, after labeling, we obtained a txt file with the same name as the image name. A picture was taken from the defective insulator dataset as an example; its content contained two parts, respectively. The first number was the category to which the labeled box belonged, and it was the defective insulator. Thus, it belonged to the first category, and since the number started from 0, the first number was also. The next four numbers were the pixel coordinates of the labeled box; for example, the first group of data showed the information of the first labeled box, [0.150625 0.553158 0.249250 0.457740]. These numbers represented the center point (x, y) of the target labeled real box and the width and height information of the labeled real box, which intuitively showed the location information of the labeled box. The position information of the marked box could be visualized.

### 3.2. Construction of a Small Sample Insulator Dataset

Since there is less insulator photo data available on the web for study, and the open-source dataset provided does not have universality, this design preprocesses the images searched on the web to construct a dataset with a larger amount of data based on inspection images. The original dataset obtained from the internet was 120 images of defective insulators and 120 images of normal insulators. Greater volumes of data lead to higher accuracy in model detection results. Thus, cropping images and performing other preprocessing operations are necessary to obtain a larger and more generalized dataset.

**Figure 2:**
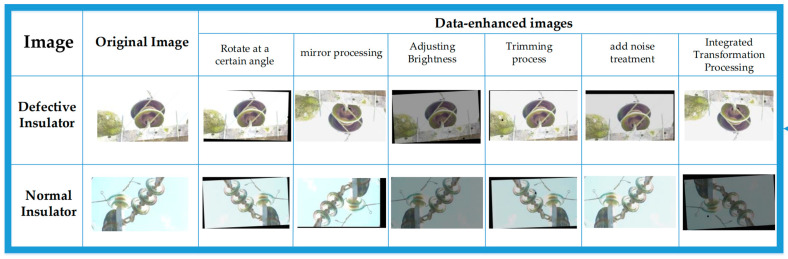
A part of the image transformation result demonstration.

To expand the dataset, we used several image processing methods. We cropped the original image to randomly remove some pixels; we also performed pixel panning, which slightly shifts pixels in the image horizontally or vertically to increase the diversity of the dataset; and to change the brightness of the image, we adjusted the background by scaling the RGB value of each pixel using a linear transform to adjust the brightness of the image to make it brighter or darker. This simulates different lighting conditions and makes the model robust to images with different brightness conditions. To increase the diversity of the dataset, we also add noise processing. By adding random noise, such as Gaussian or pretzel noise, to the images, we can simulate real-world image noise and make the model better able to cope with these disturbances. In addition, we also performed rotation and reflection processing. By setting different rotation angles, we could change the orientation of the images and increase the diversity of the dataset. At the same time, we also performed mirroring processing on some of the images, which made the images flip in horizontal or vertical directions, further increasing the richness of the dataset. By applying the above image processing methods, we obtained a new set of images to expand the dataset. Based on the original 1025 images, we extended the original 240 images into a completely new insulator dataset. This made the dataset richer and more diverse, providing a wider range of samples for training the model. Figure 2 shows the results of the partial image processing, showing the effects of the different transformations we performed on the images.

**Figure 3:**
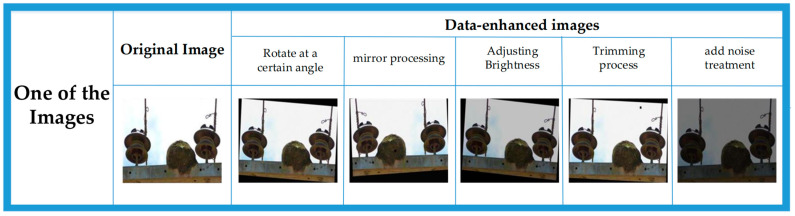
The processed image.

Taking one of the images as an example, the image after various transformations is shown in Figure 3 below.

### 3.3. Introduction to YOLOV5 and Parameter Optimization

#### 3.3.1. YOLOV5 Introduction

**Figure 4:**
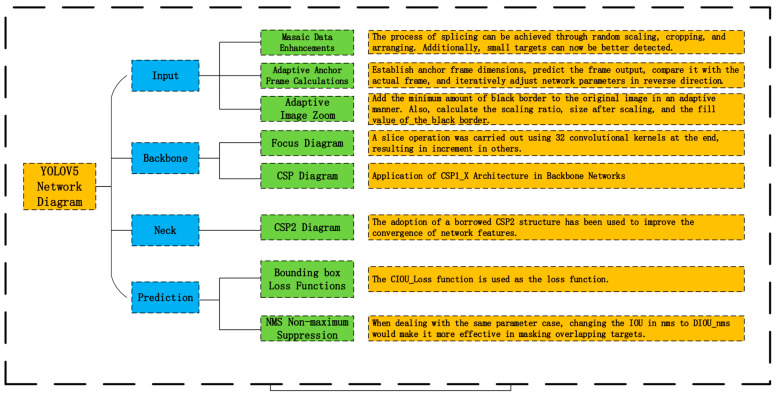
The network diagram of YOLOV5.

The YOLOV5 algorithm consists of four parts—input, backbone, neck, and prediction—and it has four types of network structures: YOLOV5s, YOLOV5m, YOLOV5l, and YOLOV5x. In this paper, we mainly use YOLOV5s as the training model for the experiments. Compared with the previous versions of the algorithm, it has better applicability and generalizability to the dataset; YOLOV5s has a great advantage in running speed compared with the other models, and the trained model and test data are more substantial than the previous algorithms. The network diagram of YOLOV5 is depicted in Figure 4.

**Figure 5:**
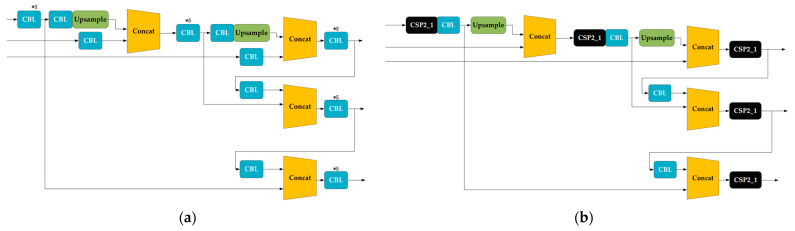
Comparison of neck structures: (**a**) the neck structure of YOLOV4; (**b**) the neck structure of YOLOV5.

There are three key improvements over YOLOV4:

(1) The focus structure is first referenced, and its key feature is the slicing operation. For instance, in YOLOV5s, with 304 × 304 × 3 image pixels, the focus structure performs the slicing operation, resulting in 152 × 152 × 12 feature maps, which undergo a single convolution operation using 32 kernels, leading to 152 × 152 × 32 feature maps.
(2) The neck structure in the YOLOV5 algorithm uses CSPnet and CSP2 structure instead of all ordinary convolution operations used in the original algorithm. This improves the neural network’s performance for feature vector fusion, as depicted in Figure 5.
(3) Improving the bounding box loss function: In YOLOV5, *CIOU_Loss* is adopted as the loss function for the bounding box. The details of YOLOV5’s loss function will be explained in the following section. The loss function L for YOLOV5 is primarily comprised of three components: the location loss function $L_{Location}$, the confidence loss function $L_{Objects}$, and the type of loss function $L_{Classes}$. The loss function L is shown in Equation (1):
  (1) $$L=L_{\mathrm{Location}}+L_{Objects}+L_{Classes}$$

The position loss function is evaluated using the CIOU_Loss, as presented in Equation (2):

(2) $$CIOU\_Loss=1-CIOU=1-(IOU-\frac{\mathrm{Distance}\_2^{2}}{\mathrm{Distance}\_C^{2}}-\frac{v^{2}}{(1-IOU)+v})$$

In the formula:

*C*—The smallest outer rectangle of the prediction frame from the actual frame is identified;

*Distance_C*—The given measure refers to the diagonal distance of the smallest outer rectangle;

*Distance*_2—The Euclidean distance between two center points can be calculated.

For the YOLOV5 algorithm, many parameters affect the behavior and performance of the algorithm. This work addresses the learning rate, cosine annealing hyperparameters, step size, number of training rounds, and other parameters to ensure that the performance of the algorithm can better meet the experimental requirements.

#### 3.3.2. Learning Rate

Learning rate is an important hyperparameter in deep learning models, as it determines the step size of the model to update the weights in each round of training. Setting a reasonable learning rate can improve the training speed and performance of the model and can better prove the generalization performance of the model. In the YOLOV5 algorithm, the learning rate is the learning rate of the model in the training process; it determines the update of the model weights in each iteration. The gradient update formula is shown in Equation (3):

(3) $$\theta =\theta -\eta \frac{\partial }{\partial \theta }\cdot J(\theta )$$

From Equation (3), it is known that if the learning rate is too high, the model will not converge during the training process and will even cause the phenomenon of gradient explosion; on the other hand, if the learning rate is too low, the model training speed will be slow and convergence will be difficult. After two training sessions with a total of 300 epochs, the learning rate is changed to 0.0032 in combination with the model, which improves the model training speed while avoiding an overfitting situation.

#### 3.3.3. Step Size

The step size is the number of pixels the convolution kernel moves each time the convolution operation is performed. Specifically, the step size is defined as the distance that the convolution kernel moves over the input image each time.

The larger the step size, the smaller the size of the output feature map, which will reduce the network’s ability to detect small targets. The larger the step size, the smaller the computational effort, which can speed up the training and inference of the model but also reduce the accuracy of the model; the smaller the step size, the more detailed information contained in the feature map extracted by the model, which can improve the expressiveness and detection accuracy of the model, but the computational effort will increase accordingly.

Therefore, the step size is generally set based on the target size, computational resources, dataset characteristics, model structure, etc. In general, it means that the step size is changed according to the target size and model structure to balance the detection accuracy and training speed of the model to achieve the optimal effect while ensuring computational capacity. This experiment was tested three times by setting the step size to 12, 8, and 7, respectively, and finally, the appropriate step size was determined to be 8.

In summary, the initial model used in this experiment is the YOLOV5s model, the depth of the model is 0.33, the width of the model is 0.50, the preset is the anchor size corresponding to 640 × 640 resolution, 200 epochs are trained, the learning rate is 0.0032, the cosine annealing hyperparameter is 0.12, the learning rate momentum is 0.843, and the step size is changed to 8 according to the experimental requirements.

### 3.4. Evaluation Indicators

In the target detection process, the main average precision (mAP) in the final model results is the main evaluation index, and the results of target detection can be divided into the following four categories according to differences, which are:

(1) True positive (TP), i.e., correct checkout results, are examples of detection results that match the true labeled results of the provided training set in the output of the network after training;
(2) True negative (TN), i.e., regions that are correct but not detected by the system, refer to the parts of the image provided for testing where the labeled insulators, bird nests, weeds, etc. are not present, and the trained network does not produce the desired detection results;
(3) False positive example (FP), i.e., false detection result, means that the test result output by the trained network does not match the true annotation of the manual annotation of the given image, which can also be called false detection;
(4) False negative (FN), or the area where the desired target is mistakenly not detected, refers to the area where targets such as insulators, bird nests, weeds, and small birds are present in the test image but are not detected by the network.

The YOLOV5 target detection algorithm often uses the IOU function to discriminate the detection results of the trained model. Each true annotation corresponds to a corresponding true positive example, which is the detection result with the highest score greater than the threshold set in the IOU, while the other corresponding detection results are false positive examples. False positives are also found when the detection results of the trained model do not match any of the provided true annotations. False positives generally include detection of other objects or image backgrounds, failure to meet the set threshold, and duplicate, useless detection results. False negatives are examples that do not match the real annotations provided by the real positive examples, generally due to insufficient accuracy or precision of detection or missed detection.

Based on the above definition, two corresponding detection metrics can be proposed: precision and recall.

The accuracy rate, which indicates the percentage of predicted results that are true among all results with positive samples, is the main indicator to evaluate the accuracy of the test results of the trained system and is defined by the formula shown in Equation (4):

(4) $$P=\frac{T_{P}}{T_{P}+F_{P}}$$

Recall indicates the percentage of all positive samples that are correctly predicted, i.e., the proportion of true positive cases among the true annotations labeled by the samples of the preselected tests to the number of all preselected test samples. This metric is the main indicator to assess the completeness of the test results of the trained system, and its defining formula is shown in Equation (5):

(5) $$R=\frac{T_{P}}{T_{P}+F_{N}}$$

Average precision (AP) is the result of integrating precision over different recall rates. Geometrically, AP is also the size of the area under the curve in the P-R curve for each category. The average precision value is generally used to measure how good or bad the trained model is for each category. The main average precision is the average of the average precision values of the different categories.

## 4. Experimental Results and Analysis

In this paper, we design a program to preprocess and annotate the existing inspection images found on the internet to form an initial, small insulator image dataset, and then use Python programming to preprocess the original images by cropping, pixel panning, changing the background brightness, adding noise, setting the rotation angle, mirroring, etc., to change the images to simulate different lighting conditions and different image quality. The insulator images under different lighting conditions and different image quality so as to realize the data expansion of the original dataset. The original small dataset consists of 240 images, the test set images are extracted from the database at a ratio of 0.025, and the remaining images are the training set, and the size of the expanded dataset is 1025. In this subsection, the insulator database is trained before and after the expanded dataset to compare and verify whether the expanded dataset is effective and the effect of the expanded dataset on the accuracy of the model trained by YOLOV5. The algorithmic network model for this experiment is YOLOV5, and the step size is set to 8. The use of hyperparameters is shown in Table 1.

**Figure 6:**
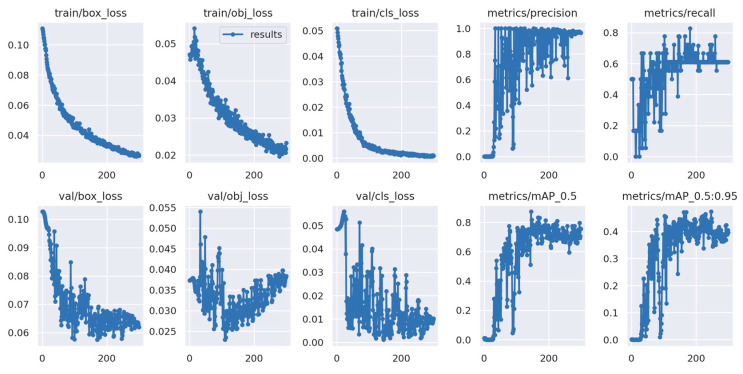
Model training process before dataset expansion.

**Figure 7:**
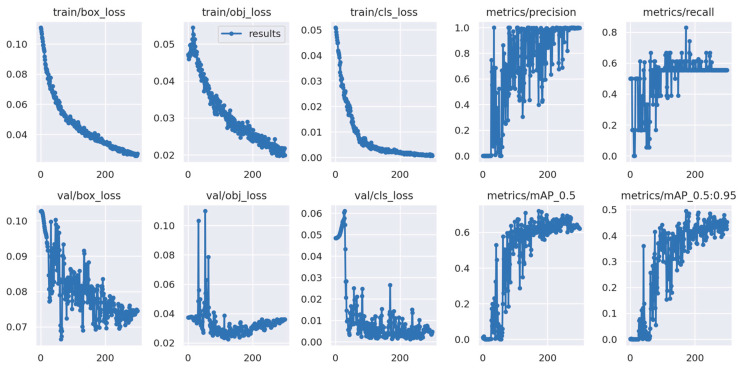
Model training process after dataset expansion.

First, a comparison of the model data after training, as shown in Table 2 and Figure 6, is the model data before the dataset expansion, and Table 3 and Figure 7 are the model data after the dataset expansion. It is obvious that the accuracy and recall of the trained model have improved significantly after the expansion of the dataset, the oscillation has improved very well, and the mAP value is 0.632 before the expansion and 0.793 after the expansion, which is a significant improvement, indicating that the trained model has been improved and optimized.

The graph above displays the meaning of each indicator as follows. box_loss: The error (CIOU) between the prediction frame and the calibration frame determines the accuracy of the positioning. A smaller error indicates better accuracy. obj_loss: The confidence level of the computational network determines the accuracy of its ability to classify a target, with lower levels indicating higher accuracy. cls_loss: This calculation determines whether the anchor frame is correctly classified in relation to the corresponding calibration. A smaller classification value indicates greater accuracy. mAP@0.5:0.95 (mAP@[0.5:0.95]): The value indicates the average mean average precision at varying intersection over union (IOU) thresholds ranging from 0.5 to 0.95 at intervals of 0.05. mAP@0.5: The statement represents the average mAP for thresholds greater than 0.5. The *y*-axis represents the number of epochs.

**Figure 8:**
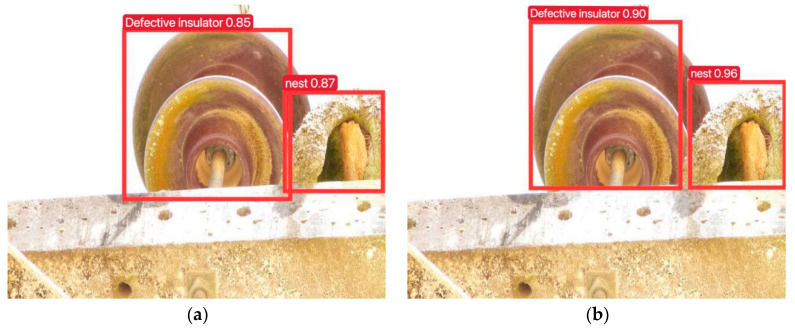
Model training before and after enriching the dataset for the detection results of defective insulators: (**a**) before enriching the dataset; (**b**) after enriching the dataset.

**Figure 9:**
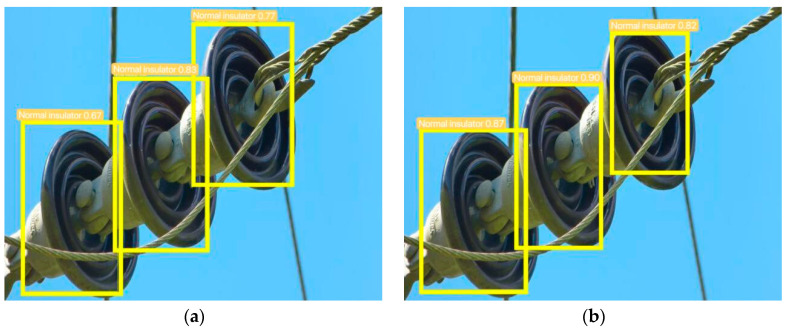
Model training before and after enriching the dataset for the detection results of normal insulators: (**a**) before enriching the dataset; (**b**) after enriching the dataset.

Two images of insulators were selected in the network to better visualize the variations between the two models; one image has defective insulators, while the other one shows normal insulators. The training model’s results for the defective insulator were captured in Figure 8 before and after the dataset expansion, and for the normal insulator, they are presented in Figure 9 before and after dataset augmentation.

For comparison purposes, a comparison of the individual data is shown in Table 4.

It can be clearly seen that whether for the detection of defective insulators, bird nests, or normal insulators, the accuracy and precision of the expanded dataset are slightly higher, which proves that the expanded dataset is effective, and the expanded dataset has improved the accuracy and precision of the training model.

## 5. Conclusions

Multisource data sensing technology is a crucial aspect of the active distribution network and essential for ensuring its safe operation. Insulators are significant equipment in transmission lines of the active distribution network and have a direct impact on the safety and reliability of the power grid’s operation. This paper proposed a detection algorithm with a higher utility rate to meet the speed and accuracy requirements of line inspection work.

(1) This paper presented an algorithm that uses YOLOV5 and a more powerful dataset for detection. The algorithm collects images associated with insulators and builds a database of insulators based on the images taken during the inspection of transmission lines provided by the network.
(2) To expand the provided small database, preprocessing operations such as image pixel cropping, rotation angle adjustment, background brightness alteration, and adding noise were performed. Through precomparison experiments and validation, it was found that the expanded dataset significantly improved the accuracy and recall rate of the training model, as well as leading to good changes in the value of the mAP. The mAP value increased from 0.632 to 0.793 after the expansion, which demonstrates a significant improvement in the trained model’s optimization. This increase in accuracy by 0.05 confirms the feasibility and applicability of the expanded dataset. It also validates that an expanded dataset can lead to better training of models and improve detection precision and accuracy.

While this paper expanded the dataset, it remains small and generally incomplete, the types of insulators in transmission lines are continuously evolving, and the types of faults are numerous. Enriching and updating the dataset will be a focus of future studies.

## Figures and Tables

**Table 1:** Hyperparameter usage statistics.

Serial Number	Hyperparameter Name	Date
entry 1	epoch	200
entry 2	Learning rate	0.0032
entry 3	Batch size	8
entry 4	Cosine annealing hyperparameters	0.12
entry 5	Learning rate momentum	0.843

**Table 2:** Model training results before dataset expansion.

Class	Images	Labels	P	R	mAP@0.5	mAP@0.5:0.95
All	6	12	0.624	0.602	0.632	0.458
Defective insulator	6	3	0.882	1	0.995	0.798
Normal insulator	6	9	0.365	0.204	0.27	0.118

**Table 3:** Model training results after dataset expansion.

Class	Image	Label	P	R	mAP@0.5	mAP@0.5:0.95
All	51	105	0.915	0.667	0.793	0.471
Defective insulator	51	26	0.831	1	0.995	0.73
Normal insulator	51	79	1	0.333	0.59	0.212

**Table 4:** Comparison of detection data before and after expanding the dataset.

Class	Label 1	Label 2	Label 3	Label 4	Label 5
Before expanding the dataset	Defective insulator 0.85	nest 0.87	normal insulator 0.67	normal insulator 0.83	normal insulator 0.77
After expanding the dataset	Defective insulator 0.90	nest 0.96	normal insulator 0.87	normal insulator 0.90	normal insulator 0.82

## Data Availability

Not applicable.
